# HDGF derived from Müller cells enhances the activation of microglia in diabetic retinopathy

**DOI:** 10.7555/JBR.38.20240386

**Published:** 2025-08-07

**Authors:** Aowang Qiu, Wenjie Yin, Ningyu Wang, Xin Wang, Qinghuai Liu, Weiwei Zhang

**Affiliations:** 1 Department of Ophthalmology, the First Affiliated Hospital of Nanjing Medical University, Nanjing, Jiangsu 210029, China; 2 Department of Endocrinology, Jiangsu Province Hospital of TCM/the Affiliated Hospital of Nanjing University of Chinese Medicine, Nanjing, Jiangsu 210029, China

**Keywords:** hepatoma-derived growth factor, diabetic retinopathy, microglia, Müller cell, inflammatory response, integrin beta 2

## Abstract

Diabetic retinopathy (DR), a common complication of diabetes, is characterized by retinal angiogenesis and inflammation. The role of hepatoma-derived growth factor (HDGF) in mediating inflammation during DR remains unclear. We measured HDGF levels in the aqueous humor and found that HDGF was increased in DR but decreased after anti-angiogenesis treatment. Using public single-cell RNA sequencing datasets, we found that elevated HDGF in DR was mainly produced by Müller cells and targeted microglia. Additionally, integrin beta 2 (*Itgb2*), a target gene of HDGF that induces microglial activation, was significantly upregulated in DR. To verify these results, we performed enzyme-linked immunosorbent assays, quantitative reverse transcription-PCR, Western blotting, and fluorescence immunostaining in cultured Müller and microglial cells treated with HDGF or anti-HDGF, as well as in DR mice receiving intravitreal injections of HDGF or its antibody. Exogenous HDGF further promoted microglial activation, migration, and secretion of pro-inflammatory cytokines, while neutralization of HDGF suppressed these effects caused by high glucose. Furthermore, the HDGF receptor nucleolin was overexpressed in microglia under high glucose stimulation. Therefore, blocking HDGF from Müller cells in DR reduced the excessive inflammatory response in microglia, highlighting HDGF as a potential therapeutic target.

## Introduction

Diabetic retinopathy (DR), a common microvascular complication of diabetes, primarily presents as retinal vascular leakage and neovascularization^[1]^. DR is categorized into two stages based on progression and severity: non-proliferative DR and proliferative DR (PDR). PDR is characterized by retinal neovascularization, a condition that severely impairs vision. Treatment options, such as panretinal photocoagulation and vitrectomy, are employed based on lesion characteristics. Given the significant elevation of vascular endothelial growth factor (VEGF)—a pro-angiogenic factor leading to the breakdown of the blood-retina barrier —in the eyes affected by DR, intravitreal anti-VEGF therapy is recommended to reduce DR-associated microvascular complications^[2]^. However, neutralizing VEGF, which is mainly derived from Müller cells, to inhibit retinal angiogenesis has limitations in some cases, implicating the complex pathogenesis of DR^[3–5]^. By analyzing cases with poor responses to anti-VEGF treatment, growing evidence suggests that loss of inflammatory control is critical for retinal dysfunction in DR^[4]^. Besides angiogenesis, inflammation also plays a role in DR development, including increased production of inflammatory molecules and activation of inflammatory cells^[6–7]^. As the resident tissue macrophages in the retina, microglial cells, marked by ionized calcium-binding adapter molecule 1 (IBA1), are involved in the inflammatory progression of DR through microglial-mediated upregulation of pro-inflammatory cytokines, such as interleukin-1 beta (IL-1β), interleukin-6 (IL-6), and tumor necrosis factor-alpha (TNF-α)^[8–9]^. Notably, integrin beta 2 (ITGB2, also known as CD18), which is highly expressed on microglia, is considered to trigger microglial activation, migration, and phagocytosis^[10–12]^. Thus, it is important to explore new target molecules related to both angiogenesis and inflammation in the progression of DR.

Hepatoma-derived growth factor (HDGF), initially purified from the supernatant of *in vitro*-cultured human hepatoma cell lines, has growth-stimulating, angiogenesis-inducing, and potentially pro-inflammatory roles^[13–15]^. Recently, Hu *et al*^[16]^ revealed that HDGF stimulated reactive oxygen species generation in hepatoma cells *via* binding to nucleolin (NCL). HDGF enhances angiogenic activity in tumors by stimulating endothelial mitogenesis and VEGF expression^[17]^. In 2016, LeBlanc *et al*^[18]^ reported that HDGF promoted the proliferation and migration of human retinal microvascular endothelial cells under normal conditions, indicating a potential angiogenic effect of HDGF in the retina. Notably, blocking HDGF attenuates inflammation caused by activated microglia in the brain, suggesting an inflammatory role of HDGF in the central nervous system^[19]^. Moreover, macrophage-specific deficiency of HDGF results in a significant reduction in inflammation and M1 macrophage content in atherosclerotic mice^[20]^. However, whether HDGF aggravates DR by activating inflammatory cells remains unclear.

In the present study, we analyzed genes identified as putative targets of HDGF in the MSigDB database (https://www.gsea-msigdb.org/gsea/index.jsp) using single-cell transcriptional data from the mouse retina and conducted both *in vivo* and *in vitro* experiments to provide a new perspective on the pro-inflammatory role of HDGF in the progression of DR.

## Materials and methods

### Ethics statement

The normal ocular tissue was obtained from a 72-year-old male donor who had no history of diabetes or any other ocular diseases at the Department of Ophthalmology, the First Affiliated Hospital of Nanjing Medical University. The extraction procedure was conducted in accordance with the methodology outlined in the relevant standards (https://ahpo.net/assets/2013_prof_251_rcophth_standards_for_the_retrieval_of_humanc_ocular_tissue_1.pdf). All procedures followed standard eye donation protocols for research and the Declaration of Helsinki, with written informed consent signed by the donor before donation. The current study was approved by the Institutional Review Board of the First Affiliated Hospital of Nanjing Medical University (Approval No. 2017-SR-283). Written informed consent was obtained from all participants.

### PDR and control cohort

Patients with PDR, a severe stage of DR, were enrolled between 2017 and 2018 at the Department of Ophthalmology, the First Affiliated Hospital of Nanjing Medical University in Nanjing, China. Patients received a 0.5-mg intravitreal injection of conbercept (Chengdu Kang Hong Biotech Co., Ltd., Chengdu, Sichuan, China), an approved anti-VEGF medication. Aqueous humor samples from PDR patients were collected before and seven days after the conbercept injection. In parallel, 12 patients with idiopathic macular holes (MHs) but no other retinal diseases were included as controls.

### Animals

All animal procedures were conducted in accordance with the ARRIVE guidelines and the NIH Guide for the Care and Use of Laboratory Animals (NIH Publication No. 86-23, revised 1996), and were approved by the Institutional Animal Care and Use Committee (IACUC) of Nanjing Medical University (Approval No. IACUC-2202030). C57BL/6J (6-week-old) male mice were purchased from the Animal Core Facility of Nanjing Medical University (Nanjing, China). Mice were housed and maintained under environmentally controlled conditions (ambient temperature, 24 ℃; humidity, 40%) with a 12-h light/dark cycle and had free access to food and water.

The induction of type 1 diabetes by streptozotocin (STZ; Cat. #18883-66-4, Sigma-Aldrich, St. Louis, MO, USA) was induced by intraperitoneal injection of an appropriate dose of the drug dissolved in sodium citrate buffer (50 mmol/L) according to previous studies^[21–22]^. The solution was prepared fresh before administration, with a pH of 4.5 and was kept at 4 ℃. This solution was then injected intraperitoneally into each pre-starved (8 h) mouse at 55 mg/kg within 15 min for five consecutive days. Animals were divided into two groups: sodium citrate buffer-injected control mice (*n* = 6) and STZ-injected mice (*n* = 6). We measured blood glucose levels using a Contour TS blood glucose meter (Bayer, Leverkusen, Germany) one week after the final injection. Only mice with a blood glucose concentration > 16.7 mmol/L were considered suitable models for further study. Mice were anesthetized by intraperitoneal injection of ketamine (80 mg/kg) and xylazine (4 mg/kg), and pupil dilation was achieved with 1% cyclopentolate hydrochloride and 2.5% phenylephrine before invasive procedures.

For intravitreal injection in mice^[23–24]^, 10-week-old healthy mice (*n* = 3) received 1 μL PBS in the left eye, and 1 μL solution containing recombinant HDGF (rHDGF, 100 μg/mL, Cat. #HY-P700181AF, MedChemExpress, Monmouth Junction, NJ, USA) in the right eye using a 33-gauge needle (Hamilton, Bonaduz, Switzerland). Similarly, age-matched DR mice (*n* = 3) received 1 μL PBS in the left eye and 1 μL solution containing anti-HDGF antibody (500 μg/mL, Cat. #11344-1-AP, Proteintech, Rosemont, IL, USA) in the right eye.

For tissue preparation, mouse eyeballs were enucleated five days post-intravitreal injection following euthanasia. To prepare cryosections, the eyeballs were fixed in a FARS eyeball fixator at room temperature for 1 h, and then dehydrated overnight in 30% sucrose. The samples were subsequently embedded in optimal cutting temperature (OCT) compound, frozen using liquid nitrogen, and sectioned in a dorsal-to-ventral orientation at a thickness of 6 μm using a Leica CM1900 cryostat (Leica). To obtain mouse retinas, the cornea, iris, and lens were removed after eyeball extraction. The retina was then carefully separated from the retinal pigment epithelium layer to preserve retinal integrity.

### Cell culture

Human retinal Müller cells were purchased from Cell Research (Cat. #PRI-H-00246, Shanghai, China) and cultured in DMEM/F12 medium (Gibco, Carlsbad, CA, USA) supplemented with 10% fetal bovine serum (FBS; Invitrogen, Carlsbad, CA, USA) and streptomycin (100 U/mL; Invitrogen) at 37 ℃, 5% CO_2_. The second passage (P2) was used in the study.

The immortalized murine microglial cell line (BV2) (Cat. #CRL-2469) was purchased from the American Type Culture Collection and cultured in DMEM/F12 medium (Gibco) supplemented with 10% FBS (Invitrogen), 1% glutamine (Invitrogen), and streptomycin (100 U/mL; Invitrogen) at 37 ℃, 5% CO_2_.

For cell treatment, 5 g/L or 1 g/L glucose in DMEM/F12 medium was used to simulate DR or non-DR environments, respectively. To confirm the inflammatory role of HDGF, rHDGF (100 ng/mL) or anti-HDGF antibody (500 ng/mL) was delivered to cultured cells.

### Cluster identification and differentially expressed gene analysis

Seurat analysis was performed in R (version 4.0.2) using Seurat (version 3.1.5), along with t-SNE (Rtsne package, version 0.15), ggplot2 (version 3.3.5), and dplyr (version 1.0.7). The mouse retina single-cell transcriptome data (GSE178121) were analyzed. We first normalized and preprocessed each dataset before analysis, which included removing low-quality cells and genes based on the following criteria: nFeature_RNA > 500, nCount_RNA > 100, nCount_RNA < 2000, and percent.mt < 20. For normalization, we employed the NormalizeData function in Seurat to mitigate the impact of sequencing depth. We then utilized the FindIntegrationAnchors function to align cell populations across datasets and generate integration anchors. To assess batch effects, we employed both visualization and statistical methods. Specifically, we visualized results using UMAP (uwot package, version 0.1.10) and t-SNE (Rtsne package, version 0.15) to determine whether cells from different batches clustered together. Additionally, we used the FindMarkers function to compare cells across batches and identify significantly differentially expressed genes. To further investigate batch effects, batch was included as a covariate in the differential expression analysis. In summary, approximately 14000 single cells from three healthy and three STZ-induced DR murine retinas were included. Datasets were integrated using Seurat (version 3.1.5) by performing canonical correlation analysis (CCA) on the combined datasets and reducing them into a lower-dimensional space using dimensionality reduction (npcs = 50). To identify HDGF-targeted differentially expressed genes in microglia between healthy and DR retinas, we applied the FindMarkers function with a threshold of |log_2_(fold change)| ≥ 0.25 and an adjusted *P*-value (Benjamini-Hochberg correction) < 0.05. To evaluate the expression of HDGF target genes across retinal cell types, we used the AddModuleScore function to assess the enrichment of the HDGF target gene set (obtained from the MSigDB database). Gene enrichment analysis was performed using clusterProfiler (version 3.18.1) to validate the reliability of our results. For differential expression analysis between cell groups, Seurat's bimod test was applied with thresholds of |log_2_(fold change)| ≥ 1.5 and adjusted *P*-value (false discovery rate [FDR]) ≤ 0.05.

### RNA isolation and quantitative reverse transcription-PCR (RT-qPCR)

Total RNA was extracted from human retinal Müller cells and BV2 cells using the RNAprep Pure Micro Kit (Cat. #DP420, TIANGEN, Beijing, China) and reverse transcribed with the PrimeScript™ RT Reagent Kit with gDNA Eraser (Cat. #RR037A, TAKARA, Beijing, China) to generate cDNA. Quantitative PCR was performed using a 7300 Real-Time PCR System (Applied Biosystems) with ChamQ Universal SYBR qPCR Master Mix (Cat. #Q711, Vazyme, Nanjing, China). Absolute expression levels of *HDGF* and *ITGB2* were calculated using standard curves. Human 18S ribosomal RNA (18S rRNA) and mouse glyceraldehyde-3-phosphate dehydrogenase (*GAPDH*) were analyzed in parallel for normalization of mRNA expression. The sequences of primers (5′ to 3′) used for RT-qPCR were as follows (all primer efficiencies were between 95% and 105%): 18S rRNA, ACCCGTTGAACCCCATTCGTGA (forward), GCCTCACTAAACCATCCAATCGG (reverse); *GAPDH*, CATCACTGCCACCCAGAAGACTG (forward), ATGCCAGTGAGCTTCCCGTTCAG (reverse); *HDGF*, CCAAAGACCTCTTCCCTTACGAG (forward), TGGTTCAGGCTCTTCCACACAG (reverse); *ITGB2*, CTTTCCGAGAGCAACATCCAGC (forward), GTTGCTGGAGTCGTCAGACAGT (reverse).

### Enzyme-linked immunosorbent assay (ELISA)

HDGF levels in the aqueous humor were measured using a double-antibody sandwich ELISA, with a mouse anti-human HDGF monoclonal antibody as the capture antibody and a rabbit anti-human HDGF polyclonal antibody as the tracer antibody in a 100 µL reaction system. Each sample was diluted fourfold with blocking buffer. Bound rabbit anti-HDGF polyclonal antibody was detected by adding alkaline phosphatase-conjugated anti-rabbit IgG (1∶2000, Cat. #A3687, Sigma-Aldrich). Optical density (OD) at 405 nm was measured using a Quant Microplate Spectrophotometer (Thermo Fisher, Carlsbad, CA, USA). All samples were tested in duplicate. Serially diluted recombinant human HDGF protein, ranging from 32 ng/mL to 0.5 ng/mL, was used to plot the standard curve and quantify the concentration of HDGF in each aqueous humor sample.

The levels of IL-1β, IL-6, and TNF-α were measured using a mouse ELISA Kit (ABclonal, Woburn, MA, USA) following the manufacturer's instructions. At 48 h after rHDGF treatment, the supernatant of BV2 cells was collected to assess IL-1β secretion. Five days after intravitreal injection, retinal lysates were collected to assess IL-1β, IL-6, and TNF-α levels.

### Immunofluorescence staining

For immunofluorescence staining, cells and retinal cryosections were incubated with primary antibodies at 4 ℃ overnight, followed by corresponding fluorophore-conjugated secondary antibodies (1∶500, Cat. #A-11001, Cat. #A-11012, Cat. #A-21247, Invitrogen) at room temperature for one hour. Cell nuclei were counterstained with DAPI (1∶100, Cat. #D9542, Sigma-Aldrich). Fluorescence was observed using an LSM 510 confocal microscope (Carl Zeiss, Oberkochen, Germany). The primary antibodies used were: rabbit anti-HDGF polyclonal (1∶500, Cat. #11344-1-AP, Proteintech), mouse anti-HDGF monoclonal (1∶500, Cat. #60064-1-Ig, Proteintech), anti-CD68 (1∶500, Cat. #ab125212, Abcam, Cambridge, MA, USA), anti-ITGB2 (1∶200, Cat. #10554-1-AP, Proteintech, Wuhan, China), anti-IBA1 (1∶500, Cat. #ab283346, Abcam, Cambridge, MA, USA), and anti-glutamine synthetase (1∶500, Cat. #ab64613, Abcam).

### Immunoblotting

For immunoblotting, cells were harvested at 48 h post-treatment, and retinal tissues were collected five days post-treatment in lysis buffer (Cat. #P0013B, Beyotime, Shanghai, China) with a protease inhibitor cocktail (Cat. #P1045, Beyotime) for protein isolation. Extracted proteins were separated by gel electrophoresis, transferred to polyvinylidene fluoride (PVDF) membranes (Millipore, Boston, MA, USA), incubated with primary antibodies, and probed with horseradish peroxidase-conjugated secondary antibodies (1∶10000, Cat. #A0208, Cat. #A0216, Beyotime). Blots were developed using the Tanon-5200 Multi Chemiluminescent Imaging System (Tanon Science & Technology, Shanghai, China). ImageJ software was used for protein quantification. The primary antibodies were: anti-HDGF (1∶3000, Cat. #11344-1-AP, Proteintech, Rosemont, IL, USA), anti-ITGB2 (1∶1000, Cat. #10554-1-AP, Proteintech, Rosemont, IL, USA), and anti-α-tubulin (1∶10000, Cat. #66031-1-Ig, Proteintech). For protein expression analysis, we normalized the data by selecting an internal reference protein as a control, comparing the expression levels of the target protein against those of the reference protein.

### Statistical analysis

Data analysis was performed using GraphPad Prism 8 (GraphPad Software, San Diego, CA, USA). All results are expressed as means ± standard deviation (SD). For normally distributed data with equal variance, significant differences were determined using Student's *t*-test (two groups) or one-way or two-way ANOVA (more than two groups). For non-normally distributed data or data with unequal variances, significant differences were determined by the non-parametric Mann–Whitney *U* test (two groups) or Kruskal–Wallis test, followed by a Bonferroni post hoc test (more than two groups). The paired Student's *t*-test was used to compare the means of the same subjects under two conditions. A *P* value < 0.05 was considered statistically significant.

## Results

### HDGF expression in human retina and changes in PDR patients with anti-VEGF therapy

The characteristics of all participants, including patients with MH or PDR, are summarized in ***Table 1***. Briefly, the normal eye donor was a 72-year-old male without diabetes or any other eye diseases. The 33 recruited PDR patients (female/male: 19/14) had a mean diabetes duration of 11.30 (± 7.47) years. Control aqueous humor samples were collected from 12 MH patients (female/male: 7/5). The age and characteristics of patients in both control and PDR groups were closely matched.

**Table 1:** Demographic characteristics of the participants

Groups	Numbers	Age (years, mean ± SD)	Sex (female/male)	Duration of DM (years, mean ± SD)
MH	12	52.17 ± 9.93	7/5	NA
PDR	33	52.18 ± 10.88	19/14	11.30 ± 7.47
PDR-7d	7	54.85 ± 5.79	4/3	7.00 ± 4.47
Abbreviations: DM, diabetes mellitus; MH, macular hole; NA, not applicable; PDR, proliferative diabetic retinopathy; PDR-7d, PDR patients whose aqueous humor HDGF levels were measured before and seven days after intravitreal anti-VEGF (conbercept) injection; SD, standard deviation.				

**Figure 1:**
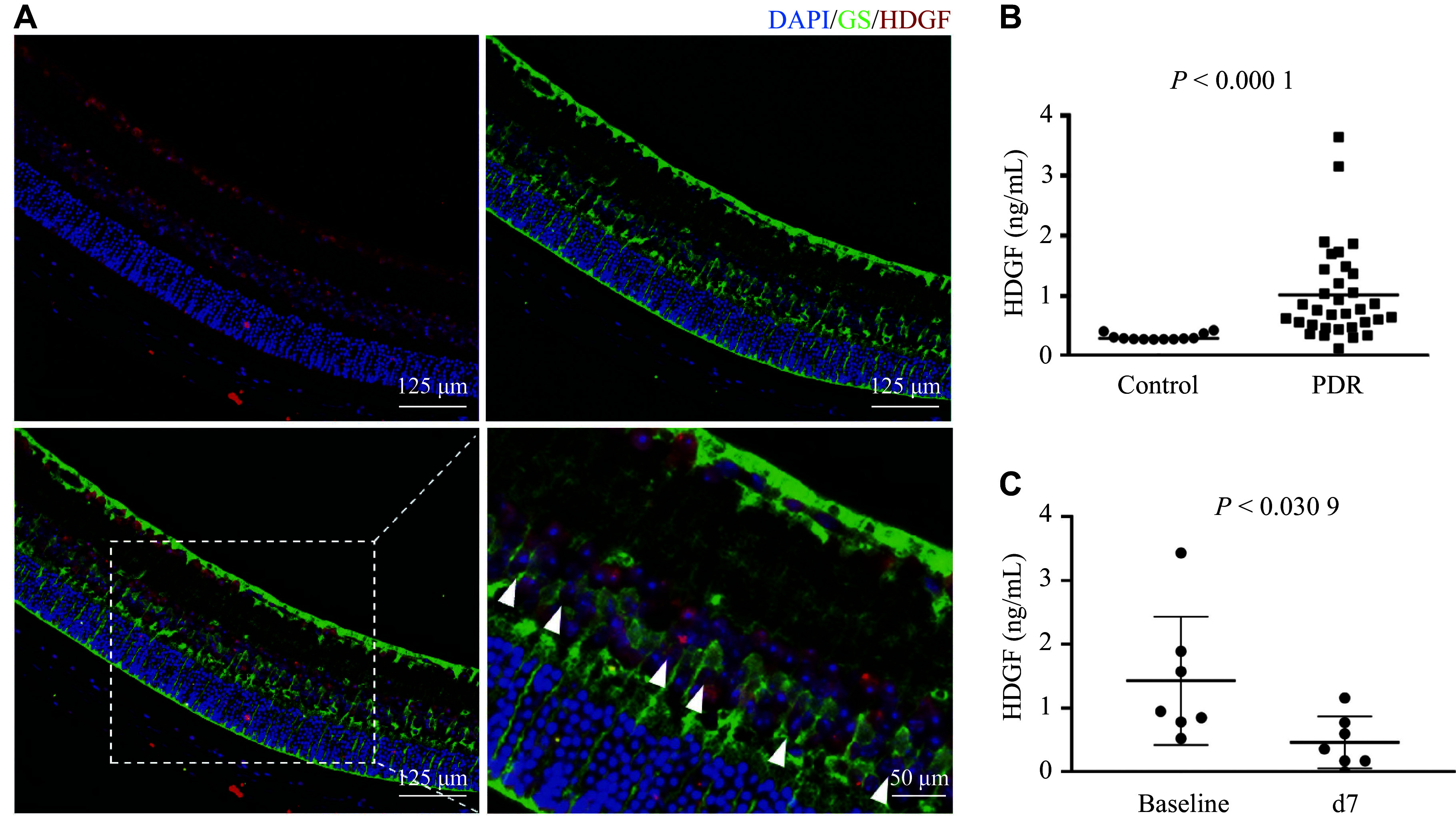
HDGF expression in the human retina and its alteration in response to anti-VEGF therapy in PDR patients. A: Representative immunofluorescence images of a normal human retinal section co-stained for HDGF (red) and the Müller cell marker glutamine synthetase (GS, green). Nuclei were counterstained with DAPI (blue). Arrowheads indicate cells positive for both markers. B: Enzyme-linked immunosorbent assay measuring HDGF protein concentration in the aqueous humor of patients. Comparison between control subjects and patients diagnosed with PDR. C: HDGF levels in PDR patients before (Baseline) and seven days (d7) after intravitreal injection of the anti-VEGF antibody (conbercept). Data are presented as individual data points with mean ± standard deviation. Statistical significance was determined by the paired Student's *t*-test. Abbreviations: DAPI, 4',6-diamidino-2-phenylindole; GS, glutamine synthetase; HDGF, hepatoma-derived growth factor; PDR, proliferative diabetic retinopathy; VEGF, vascular endothelial growth factor.

To detect the expression and distribution of HDGF in the human retina, we performed co-immunofluorescence staining of HDGF and glutamine synthetase (GS, a Müller cell marker). Strong red fluorescence for HDGF was observed in the inner nuclear layer (INL) and colocalized with GS (***Fig. 1A***).

As shown in ***Fig. 1B***, HDGF levels measured by ELISA were significantly increased in the aqueous humor of PDR patients compared with those from the MH group (1.019 ± 0.781 ng/mL *vs.* 0.318 ± 0.054 ng/mL, *P* < 0.0001). In PDR patients, HDGF levels in the aqueous humor decreased from 1.019 ± 0.781 ng/mL at baseline to 0.527 ± 0.446 ng/mL (*P* = 0.0309) seven days after intravitreal anti-VEGF (conbercept) injection (***Fig. 1C***).

### Increased HDGF production in Müller cells by high glucose stimulation

**Figure 2:**
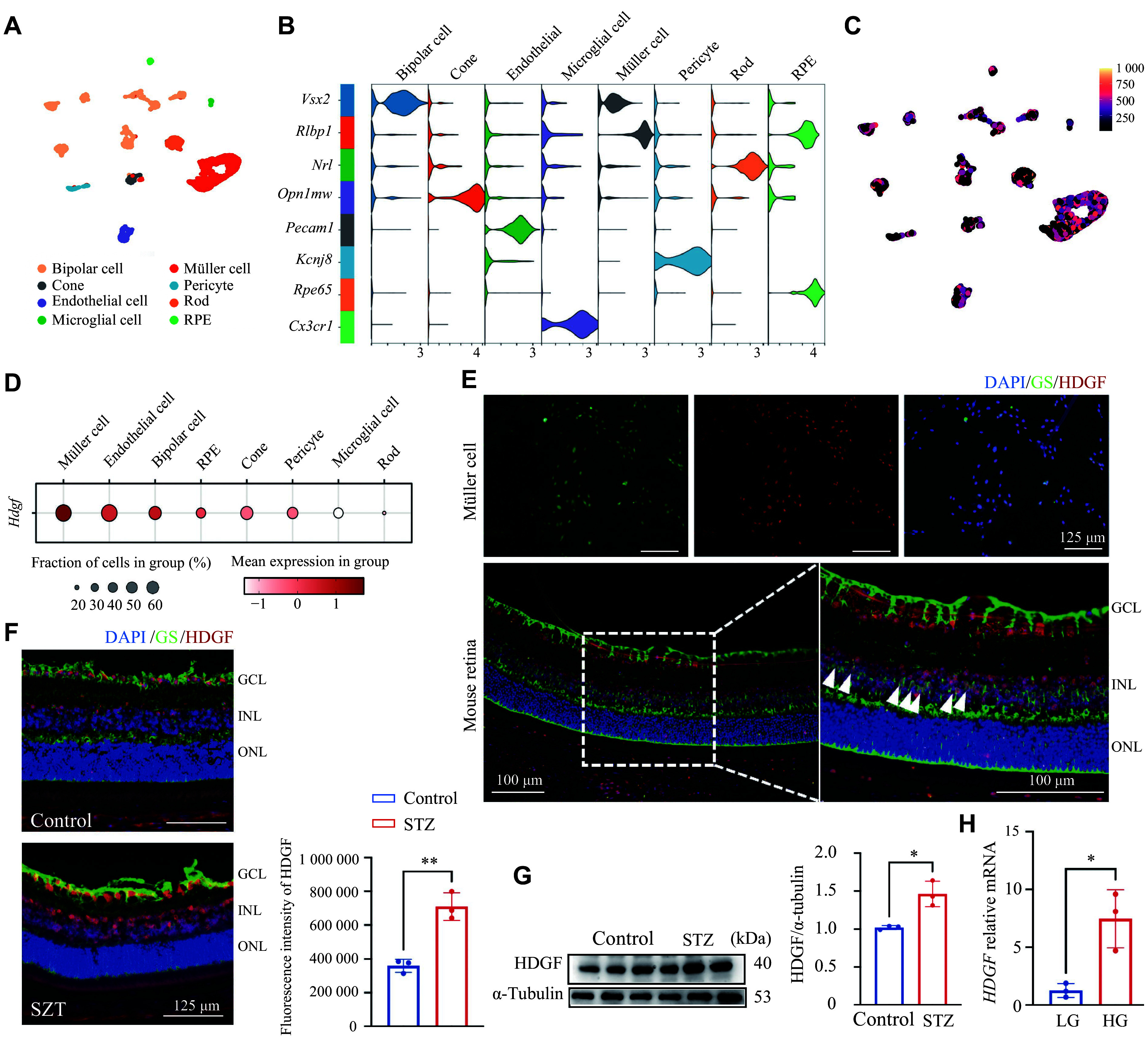
Increased HDGF production in Müller cells by high glucose stimulation. A: UMAP plot of mouse retina scRNA-seq data showing cell types. B: Violin plot of specific gene expression in different cell types. C: Feature plot visualizing *Hdgf* transcript expression levels across all cells. D: Dot plot illustrating the relative expression level and proportion of *Hdgf*-expressing cells within each cluster. E: Immunofluorescence co-staining for HDGF (red) and the Müller cell marker glutamine synthetase (GS, green) in cultured primary Müller cells and mouse retinal cryosections. Nuclei were counterstained with DAPI (blue). Arrowheads indicate representative double-positive cells. F: Representative immunofluorescence images of retinal cryosections stained for HDGF (red) and GS (green) from control mice and streptozotocin (STZ)-induced diabetic retinopathy (DR) mice at four weeks post-diabetes onset. Quantitative fluorescence intensity of HDGF in the control and DR retina (*n* = 3 mice per group). G: Western blotting analysis of HDGF protein levels in retinal lysates from control and STZ-induced diabetic mice. α-Tubulin served as the loading control. Quantitative protein levels of HDGF in the control and DR retina (*n* = 3 mice per group). H: Quantitative reverse transcription-PCR analysis of *HDGF* mRNA expression in primary Müller cells treated with low-glucose (LG, 1 g/L) or high-glucose (HG, 5 g/L) medium for 48 h (*n* = 3). Data are presented as mean ± standard deviation. Statistical significance was determined by the paired Student's *t*-test. ^*^P < 0.05 and ^**^P < 0.01. Abbreviations: GCL, ganglion cell layer; HDGF, hepatoma-derived growth factor; INL, inner nuclear layer; ONL, outer nuclear layer; STZ, streptozotocin.

To determine which retinal cells produce HDGF, we analyzed single-cell transcriptome data of mouse retinal cells obtained from a public database (GSE178121) and found that HDGF was expressed in all cell types, consistent with our human retinal staining; however, the highest expression was in Müller cells, with relatively low levels in microglia (***Fig. 2A***–***2D***). To confirm this finding, we performed co-immunofluorescence staining of GS (Müller cell marker) and HDGF in cultured retinal Müller cells and mouse retinal sections, revealing co-localization of GS and HDGF in both cultured Müller cells and retinal sections (***Fig. 2E***).

Additionally, retinal sections stained for GS and HDGF showed increased HDGF levels in STZ-induced diabetic mice compared with control mice (***Fig. 2F***). Elevated protein expression of retinal HDGF was also observed in STZ mice (***Fig. 2G***). Furthermore, high-glucose (HG) treatment for 48 h in Müller cells significantly increased *HDGF* mRNA levels (***Fig. 2H***).

### HDGF promotes microglia activation and IL-1β secretion

**Figure 3:**
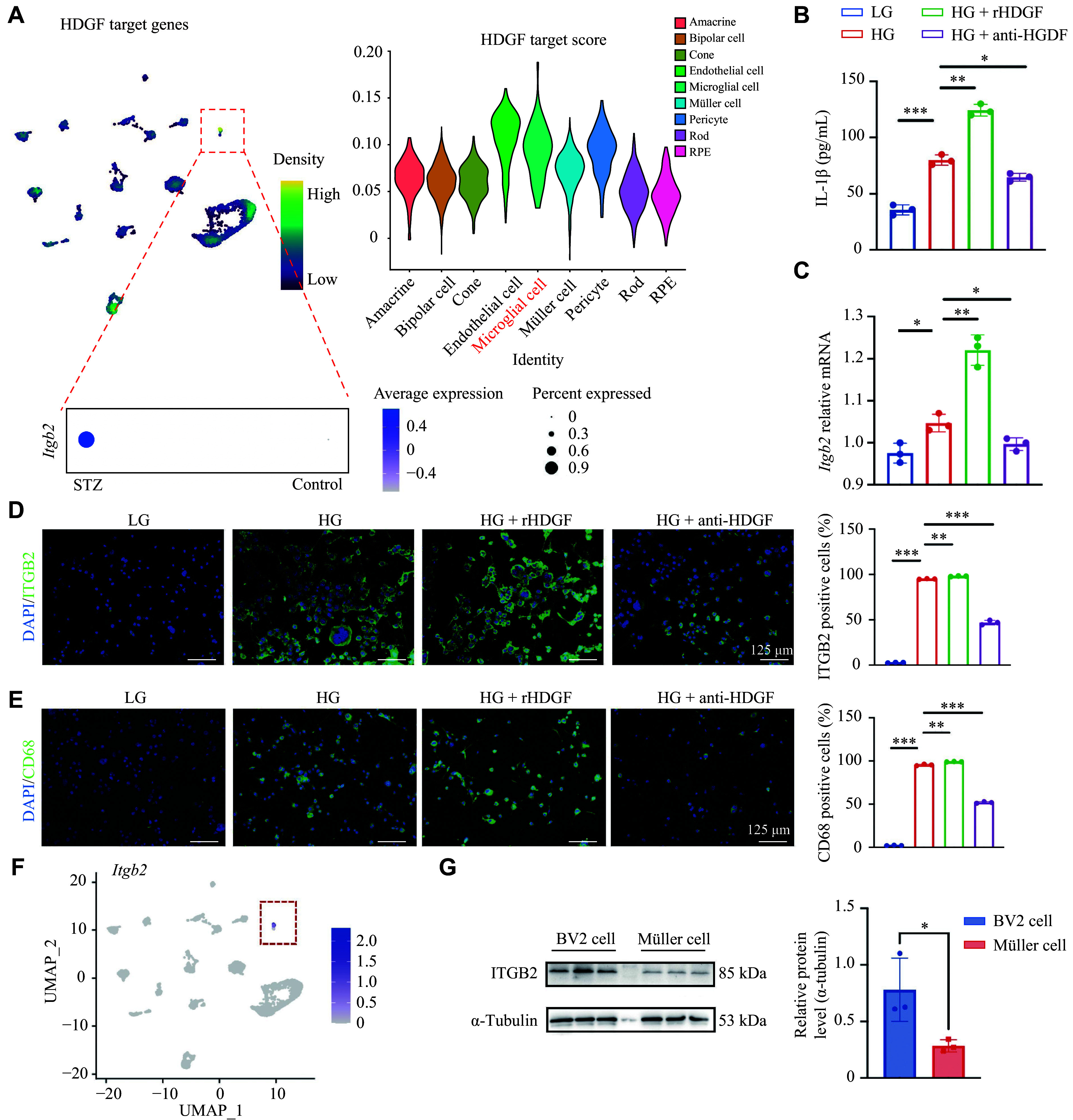
HDGF promoted microglia activation and IL-1β secretion. A: Analysis of a published list of target genes of HDGF integrated with mouse retinal single-cell RNA sequencing data, indicating predominant target cell types. The violin plot shows expression of the target gene *Itgb2* in microglia from control and streptozotocin (STZ)-induced diabetic mouse retinas. The microglia cluster is indicated. B: Enzyme-linked immunosorbent assay measuring IL-1β secretion in the supernatant of BV2 microglial cells. Cells were treated under low-glucose (LG, 1 g/L) or high-glucose (HG, 5 g/L) conditions for 48 h, with or without recombinant HDGF (rHDGF) and an HDGF-neutralizing antibody (anti-HDGF) (*n* = 3). C: Quantitative PCR analysis of *Itgb2* mRNA expression in BV2 cells treated as indicated (*n* = 3). D: Representative immunofluorescence images (left) and quantification (right) of ITGB2-positive BV2 cells following the treatments. Nuclei were counterstained with DAPI. Quantitative analysis of ITGB2-positive cells treated as indicated (*n* = 3). E: Representative immunofluorescence images (left) and quantification (right) of CD68-positive BV2 cells following the treatments. Nuclei were counterstained with DAPI. Quantitative analysis of CD68-positive cells treated as indicated (*n* = 3). F: Feature plot from retinal scRNA-seq data showing specific *Itgb2* expression within the microglia cluster (outlined). G: Western blotting analysis of ITGB2 protein expression in BV2 microglial cells and primary Müller cells. α-Tubulin served as the loading control. Quantitative protein levels of ITGB2 in BV2 and Müller cells (*n* = 3 per group). Data are presented as mean ± standard deviation. Statistical significance was determined by one-way analysis of variance (ANOVA; B–E) or paired Student's *t*-test (G). ^*^P < 0.05; ^**^P < 0.01; ^***^P < 0.001. Abbreviations: HDGF, hepatoma-derived growth factor; ITGB2, integrin beta 2.

We integrated and compared the HDGF target gene set in the MSigDB database (https://www.gsea-msigdb.org/gsea/index.jsp) with single-cell transcriptional data of mouse retina, and found that the target cells of HDGF were microglial and endothelial cells (***Fig. 3A***). In addition, *Itgb2*, a target gene of HDGF that promotes microglia activation, was upregulated in microglia of STZ mice (***Fig. 3A***). Because LeBlanc *et al*^[18]^ showed that HDGF regulated the proliferation, migration, and permeability of retinal endothelial cells, we focused on its other target cell: microglia.

BV2 microglial cells cultured under HG conditions were treated with HDGF or anti-HDGF for 48 h, with low-glucose (LG) or HG conditions alone serving as controls. HDGF further promoted the secretion of inflammatory factor IL-1β in BV2 cells under HG conditions, whereas the HDGF antibody reversed this effect (***Fig. 3B***). Importantly, both the mRNA expression (***Fig. 3C***) and immunofluorescence (***Fig. 3D***) of *Itgb2* increased with additional HDGF treatment in HG microglia and decreased with anti-HDGF treatment. Similarly, HDGF exacerbated HG-induced microglia activation as indicated by CD68, while the HDGF neutralization attenuated this activation (***Fig. 3E***).

To clarify the expression of *Itgb2* in microglia, we analyzed the single-cell transcriptional data of the mouse retina. UMAP analysis demonstrated specific *Itgb2* expression in microglia (***Fig. 3F***), consistent with the differential protein expression of ITGB2 in BV2 and Müller cells cultured under HG conditions (***Fig. 3G***).

### High glucose enhances the expression of HDGF receptor in microglia

**Figure 4:**
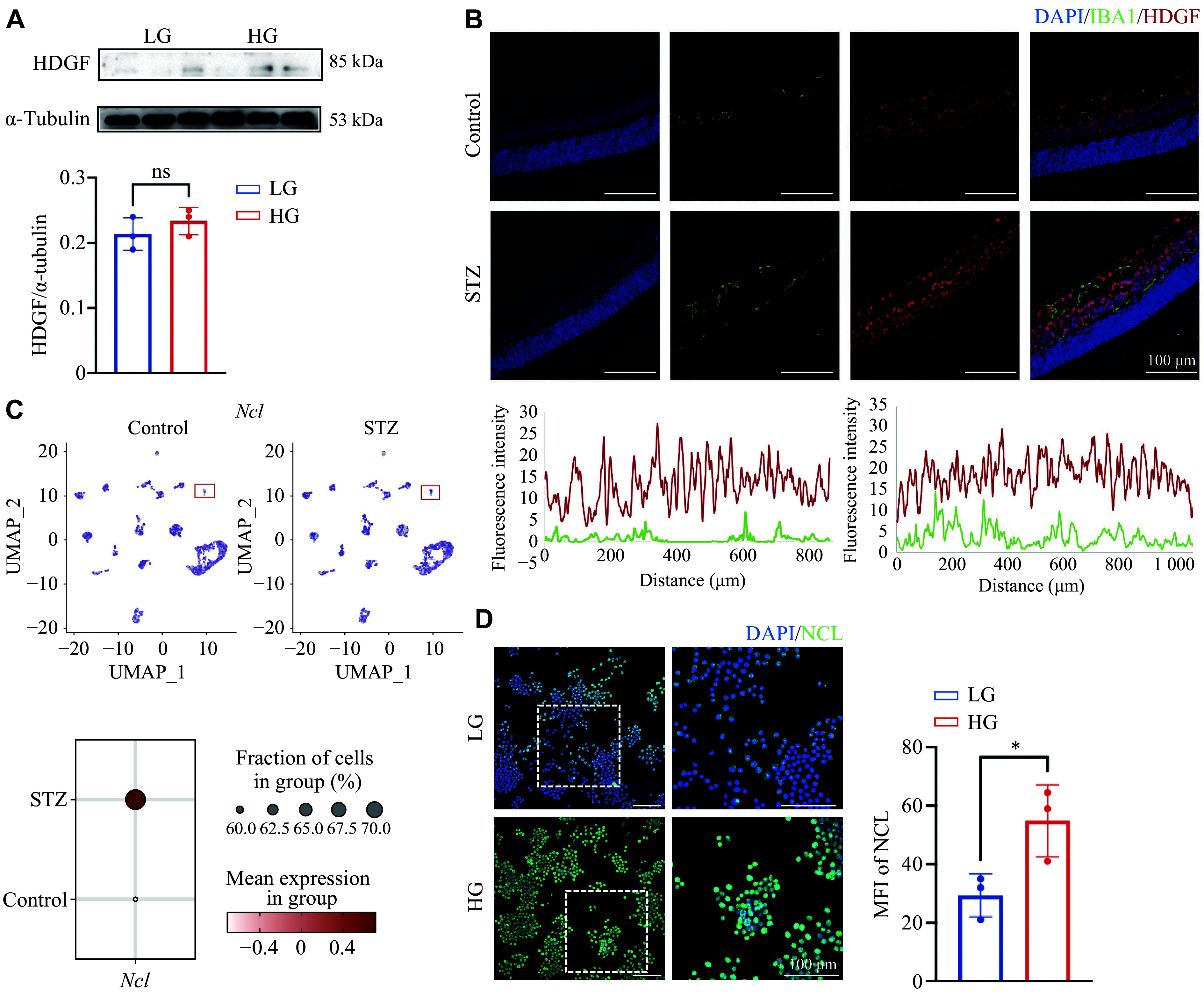
High glucose upregulated the expression of HDGF receptor in microglia. A: Western blotting analysis of HDGF protein expression in BV2 microglial cells cultured under low-glucose (LG, 1 g/L) or high-glucose (HG, 5 g/L) conditions for 48 h. α-Tubulin served as the loading control. Quantitative protein levels of ITGB2 in BV2 and Müller cells (*n* = 3 per group). B: Representative immunofluorescence images of retinal cryosections from control and streptozotocin (STZ)-induced diabetic mice, co-stained for HDGF (red) and the microglial marker IBA1 (green). Nuclei were counterstained with DAPI (blue). Assessment of immunofluorescence colocalization of HDGF and IBA1 in the two experimental groups (left, control; right, STZ). C: Single-cell RNA sequencing analysis of control and STZ-diabetic mouse retinas. UMAP plot showing the expression of *Ncl* (the gene encoding nucleolin) across all cells (upper). The microglia cluster is indicated. Dot plot illustrating the relative expression level and proportion of *Ncl*-expressing cells in microglia in the control and STZ groups (lower). D: Representative immunofluorescence images (left) and quantification of mean fluorescence intensity (MFI, right) for NCL (green) in BV2 cells cultured under LG or HG conditions for 48 h. Nuclei were counterstained with DAPI (blue). *n* = 3. Data are presented as mean ± standard deviation. Statistical significance was determined by the paired Student's *t*-test. ^*^P < 0.05. Abbreviations: HDGF, hepatoma-derived growth factor; IBA1, ionized calcium-binding adaptor molecule 1; ns, not significant; STZ, streptozotocin.

Consistent with the analysis of the public single-cell RNA-seq dataset (GSE178121), microglia showed minimal HDGF expression, as indicated by Western blot analysis of HDGF in BV2 microglia exposed to different glucose concentrations (***Fig. 4A***) and the rare colocalization of HDGF and IBA1, a microglial marker, in retina from both control and STZ mice (***Fig. 4B***). Although microglia expressed little HDGF, HDGF target genes, such as *Itgb2*, were highly expressed, suggesting that exogenous HDGF may bind to its receptor on microglia to induce transcription.

Notably, overexpression of NCL, a reported HDGF receptor in microglia, was demonstrated in STZ-induced DR mice by analysis of the public dataset (***Fig. 4C***). Similarly, increased NCL fluorescence intensity was observed in BV2 cells cultured under HD conditions (***Fig. 4D***). These results were consistent with the report by Chen *et al*^[25]^, showing that exogenous HDGF induces membrane-associated NCL accumulation in target cells, thereby binding to NCL and triggering transcription of downstream target genes.

### Neutralizing HDGF suppressed microglia activation and migration *in vivo*

**Figure 5:**
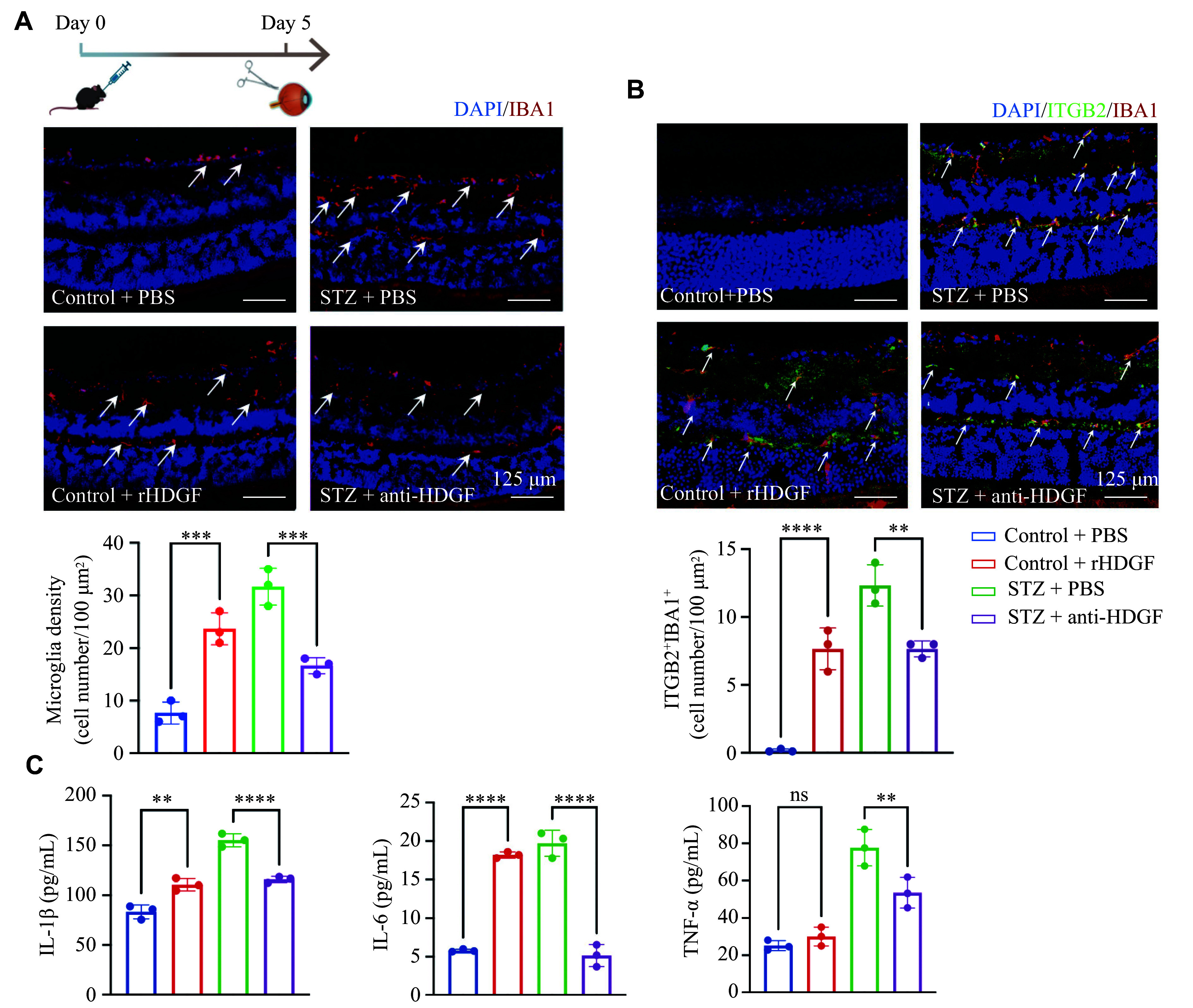
Neutralizing HDGF suppressed microglia activation and migration *in vivo*. A: Representative immunofluorescence images (left) and quantification (right) of IBA1-positive microglia (red) in retinal cryosections from the control and streptozotocin (STZ)-induced diabetic mice at four weeks post-diabetes onset. Additional images and quantification show the effect of intravitreal injection of recombinant HDGF (rHDGF) or an HDGF-neutralizing antibody (anti-HDGF) on IBA1^+^ microglia distribution and number in diabetic retinas at five days post-injection. PBS served as the vehicle control. Arrows indicate representative migrating microglia. Nuclei were counterstained with DAPI (blue). B: Representative immunofluorescence images (up) and quantification (down) of ITGB2-positive activated microglia (green) in retinal cryosections from the indicated experimental groups. Arrows indicate representative ITGB2^+^ microglia. C: Enzyme-linked immunosorbent assay measuring the levels of pro-inflammatory cytokines (IL-1β, IL-6, and TNF-α) in retinal homogenates from the indicated experimental groups. Data are presented as mean ± standard deviation (*n* = 3 per group). Statistical significance was determined by one-way analysis of variance (ANOVA). ^**^P < 0.01, ^***^P < 0.001, and ^****^P < 0.0001. Abbreviations: HDGF, hepatoma-derived growth factor; IBA1, ionized calcium-binding adaptor molecule 1; ITGB2, integrin beta 2; ns, non-significant; PBS, phosphate-buffered saline; STZ, streptozotocin.

Intravitreal injection of HDGF aggravated microglia migration (indicated by arrows in ***Fig. 5A***) to the ganglion cell layer (GCL) and outer plexiform layer (OPL) five days post-injection. However, this migration, previously reported in studies investigating the role of microglia in DR^[26–27]^, was suppressed by administration of a neutralizing anti-HDGF antibody (***Fig. 5A***). The number of IBA1^+^ retinal microglia was increased in STZ-induced diabetic mice compared with controls (***Fig. 5A***). Compared with control mice, the number of activated microglia, represented by ITGB2^+^ cells, was significantly higher in STZ mice and in mice receiving exogenous HDGF, whereas treatment with the neutralizing HDGF antibody reversed this effect (***Fig. 5B***).

Furthermore, ELISA revealed increased levels of IL-1β and IL-6 in the retinas of STZ mice and those receiving intravitreal HDGF injection, while HDGF neutralization significantly reduced the levels of these cytokines (***Fig. 5C***). Notably, HDGF injection alone did not increase the TNF-α levels; however, anti-HDGF treatment downregulated TNF-α expression in the retinas of STZ mice (***Fig. 5C***).

## Discussion

HDGF is widely expressed in embryos, normal tissues, and tumors, where it promotes organ development, cell proliferation, and angiogenesis^[28–31]^. The expression levels of HDGF in malignant tissues are significantly higher than those in normal tissues^[31]^. Serum HDGF concentration has been reported to serve as a negative prognostic marker for cancer^[32]^. Research by LeBlanc *et al*^[18]^ showed that exogenous HDGF induced the proliferation, migration, and permeability of retinal endothelial cells under normal glucose conditions. However, the role of HDGF in DR has not been thoroughly investigated.

In the current study, we measured HDGF level in the aqueous humor of patients with DR and MH and found that HDGF was significantly increased in DR samples, as expected. To identify the source of HDGF in DR, we analyzed transcriptome data from mouse retinal single cells obtained from a public database, indicating that the primary source of HDGF is Müller cells. This finding was further confirmed by co-immunofluorescence staining of GS (Müller cell marker) and HDGF in both cultured Müller cells and retinal sections. Previous studies demonstrated that the expression and function of HDGF are largely conserved between mice and humans^[13]^. Specifically, the coding sequence of HDGF shows a high degree of similarity between mice and humans, exhibiting approximately 90% identity in the amino acid sequence. During DR progression, Müller cells contribute to neuronal death, glutamate metabolism dysfunction, and the production of pro-angiogenic factors, including VEGF and pigment epithelium-derived factor (PEDF), as well as to the establishment of a chronic inflammatory retinal environment^[3,33]^. As Müller cells interact with almost every cell type in the retina, they communicate extensively with neighboring cells by secreting various factors *via* exosomes and other signaling mechanisms in DR^[34–35]^.

We integrated and compared the HDGF target gene set with the single-cell transcriptomic data from the mouse retina and found that the main target cells of HDGF are microglia and endothelial cells. Notably, the HDGF target gene *Itgb2*, representing the activation of microglia^[12,36]^, was upregulated in STZ mice. *Itgb2* has been reported to reinforce the inflammatory microglial subtype by JAK1/STAT3/IL-6 signaling^[37]^. Recently, NCL was identified as an HDGF receptor^[16]^, and exogenous HDGF stimulation increased NCL expression, enhancing HDGF uptake^[38]^. NCL is generally expressed on the cell surface as a receptor for various viruses, some bacteria, and other molecules involved in regulating inflammatory response^[39]^. We found that NCL was overexpressed in the diabetic retina, especially in microglia, indicating that excessive HDGF induced by DR may act on microglia by binding to NCL. As the immune cells in the retina, microglia are widely reported to become activated, leading to the release of pro-inflammatory molecules, microvascular damage, and neurodegeneration, contributing to DR development^[26,40–41]^. In addition, increased levels of IL-1β and IL-6 were observed in cultured microglia supernatants after HDGF exposure or in STZ mouse retinas, while anti-HDGF treatment reversed these effects. These results indicate that HDGF is directly involved in microglia activation. Interestingly, injection of rHDGF or anti-HDGF had a greater effect on IL-6 than on TNF-α and IL-1β. One possible explanation is that *Itgb2*, the HDGF target gene, directly regulates IL-6 expression^[37]^. Recently, Chung *et al*^[19]^ reported that an rHDGF antibody inhibited the upregulation of pro-inflammatory factors, including TNF-α and IL-1β, *via* NFκB signaling in the brain. NF-κB is a nuclear transcription factor that regulates microglia and Müller cell activation during DR^[42–43]^. In the current study, we utilized single-cell data from STZ models lasting over 20 weeks, while our animal model involved less than five weeks of diabetes. Although low-dose STZ models provide valuable insights into early diabetic changes, their limitations in replicating the chronicity and severity of diabetes-related complications underscore the need for caution when extrapolating findings to long-term DR. Future studies are needed to elucidate the mechanisms by which HDGF accelerates microglial inflammation during chronic DR.

Many studies have demonstrated a positive correlation between HDGF and VEGF in different cancers^[44–46]^. We also found that HDGF levels were reduced in the aqueous humor of DR patients after intravitreal injection of conbercept, a VEGF inhibitor. According to some reports^[44,46]^, HDGF induces VEGF-dependent angiogenesis by activating HIF-1α in cancers. Furthermore, VEGF stimulation in retinal endothelial cells leads to RhoA/ROCK activation^[47]^, and this activation contributes to HDGF overexpression^[48]^. Thus, VEGF inhibitors may suppress the HDGF increase *via* the RhoA/ROCK pathway. By uptaking the excitotoxins such as glutamate and secreting glutathione, Müller cells maintain metabolic homeostasis^[49–50]^. Besides the anti-angiogenic effects, conbercept treatment also elevated extracellular glutathione and alleviated inflammation in DR, indicating improved Müller cell function and retinal microenvironment^[51–52]^. This may further explain the decrease in Müller cell-derived HDGF after conbercept injection.

## Conclusion

**Figure 6:**
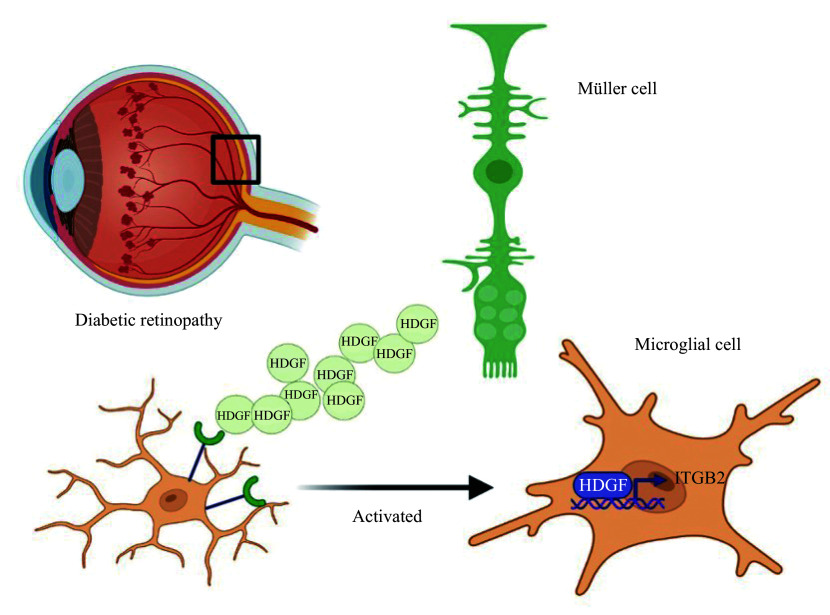
Müller cell-derived HDGF promotes microglial activation in diabetic retinopathy. HDGF secreted by Müller cells upregulates ITGB2 expression in microglia, thereby exacerbating local retinal inflammation and accelerating disease progression. Abbreviations: HDGF, hepatoma-derived growth factor; ITGB2, integrin beta-2.

In DR, increased HDGF derived from Müller cells induces microglial activation, migration, and production of inflammatory factors by enhancing *Itgb2* transcription (***Fig. 6***). Moreover, blocking HDGF in DR reduces the inflammatory response of microglia, thereby improving the retinal microenvironment and providing a novel therapeutic target for DR.
